# Conversational Agents in Health Care: Expert Interviews to Inform the Definition, Classification, and Conceptual Framework

**DOI:** 10.2196/50767

**Published:** 2023-11-01

**Authors:** Laura Martinengo, Xiaowen Lin, Ahmad Ishqi Jabir, Tobias Kowatsch, Rifat Atun, Josip Car, Lorainne Tudor Car

**Affiliations:** 1 Lee Kong Chian School of Medicine Nanyang Technological University Singapore Singapore Singapore; 2 Future Health Technologies Programme Campus for Research Excellence and Technological Enterprise Singapore-ETH Centre Singapore Singapore; 3 Institute for Implementation Science in Health Care University of Zurich Zurich Switzerland; 4 School of Medicine University of St.Gallen St.Gallen Switzerland; 5 Centre for Digital Health Interventions Department of Management, Technology, and Economics ETH Zurich Zurich Switzerland; 6 Department of Global Health and Population Harvard T.H. Chan School of Public Health Harvard University Cambridge, MA United States; 7 Centre for Population Health Sciences Lee Kong Chian School of Medicine Nanyang Technological University Singapore Singapore Singapore; 8 Department of Primary Care and Public Health School of Public Health Imperial College London London United Kingdom

**Keywords:** conceptual framework, conversational agent, chatbot, mobile health, mHealth, digital health, expert interview, mobile phone

## Abstract

**Background:**

Conversational agents (CAs), or chatbots, are computer programs that simulate conversations with humans. The use of CAs in health care settings is recent and rapidly increasing, which often translates to poor reporting of the CA development and evaluation processes and unreliable research findings. We developed and published a conceptual framework, designing, developing, evaluating, and implementing a smartphone-delivered, rule-based conversational agent (DISCOVER), consisting of 3 iterative stages of CA design, development, and evaluation and implementation, complemented by 2 cross-cutting themes (user-centered design and data privacy and security).

**Objective:**

This study aims to perform in-depth, semistructured interviews with multidisciplinary experts in health care CAs to share their views on the definition and classification of health care CAs and evaluate and validate the DISCOVER conceptual framework.

**Methods:**

We conducted one-on-one semistructured interviews via Zoom (Zoom Video Communications) with 12 multidisciplinary CA experts using an interview guide based on our framework. The interviews were audio recorded, transcribed by the research team, and analyzed using thematic analysis.

**Results:**

Following participants’ input, we defined CAs as digital interfaces that use natural language to engage in a synchronous dialogue using ≥1 communication modality, such as text, voice, images, or video. CAs were classified by 13 categories: response generation method, input and output modalities, CA purpose, deployment platform, CA development modality, appearance, length of interaction, type of CA-user interaction, dialogue initiation, communication style, CA personality, human support, and type of health care intervention. Experts considered that the conceptual framework could be adapted for artificial intelligence–based CAs. However, despite recent advances in artificial intelligence, including large language models, the technology is not able to ensure safety and reliability in health care settings. Finally, aligned with participants’ feedback, we present an updated iteration of the conceptual framework for health care conversational agents (CHAT) with key considerations for CA design, development, and evaluation and implementation, complemented by 3 cross-cutting themes: ethics, user involvement, and data privacy and security.

**Conclusions:**

We present an expanded, validated CHAT and aim at guiding researchers from a variety of backgrounds and with different levels of expertise in the design, development, and evaluation and implementation of rule-based CAs in health care settings.

## Introduction

### Background

Conversational agents (CAs), or chatbots, are broadly defined as computer programs that simulate conversations with humans [1-3]. Although the terms CA and chatbot are often used interchangeably [1,4], they sometimes define distinct conversational systems. For example, the term chatbot generally defines text-based dialogue systems and may also be used to define dialogue systems engaging in informal conversations without a specific purpose [5,6]. CAs may communicate using a variety of input-output modalities, such as text, speech, or multimedia, and adopt diverse personalities, such as coach, peer, and expert. Given this diversity, CAs can be classified according to various dimensions, such as their purpose [1], delivery channel [1], input and output modalities [1], or the response generation model [7]. Thus, enhanced clarity about the definition and classification of health care CAs is needed to understand their scope and leverage their capabilities in health care settings, from user-initiated interventions for the self-management of chronic conditions to supporting patient-provider communication.

CAs are increasingly used in health care settings for patient education [8], triage and diagnosis [9,10], and delivery of physical and mental health interventions [11,12]. CAs may alleviate health care providers’ burden by advising on the initial management of a specific complaint [13] or assisting in chronic disease management [14,15]. In addition, they can supplement providers’ care in hybrid health care delivery models [16]. Most health care CAs follow a rule-based approach, offering developers full control over the conversation flow and the information provided [17]. In health care settings, rule-based CAs may reduce miscommunication and the risk of harm arising from inappropriate advice or inaccurate triaging [2,18].

### Designing, Developing, Evaluating, and Implementing a Smartphone-Delivered, Rule-Based CA Conceptual Framework

**Figure 1:**
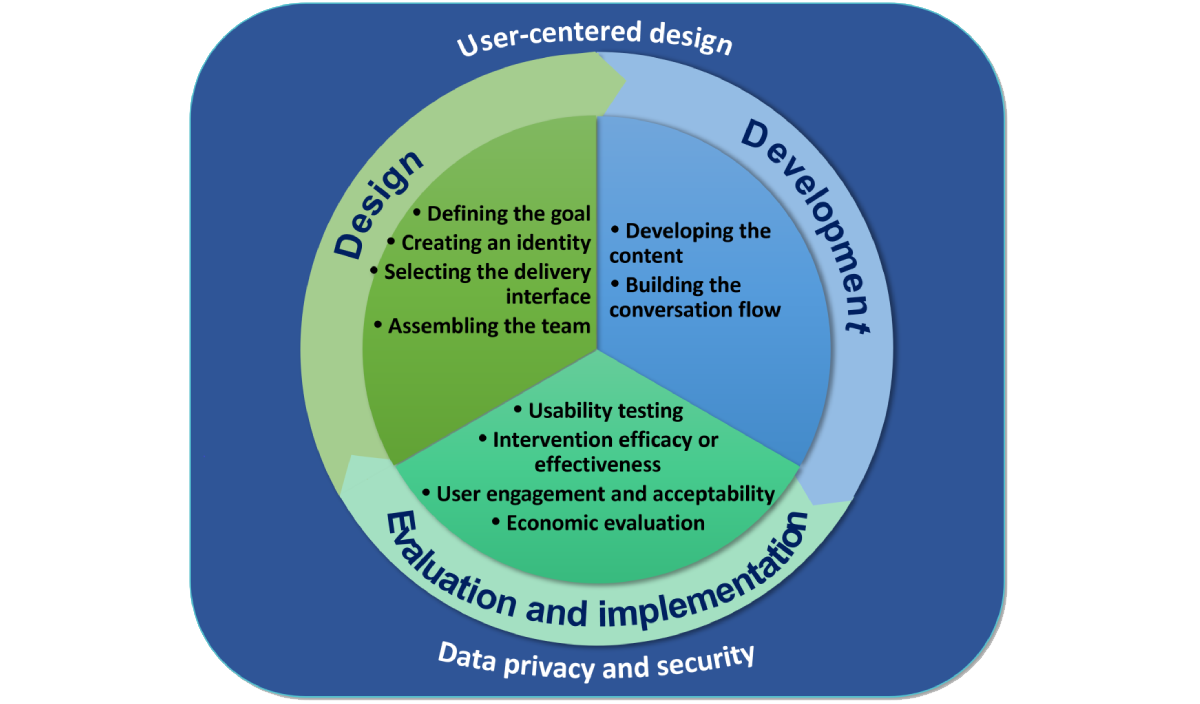
Conceptual framework for development, evaluation, and implementation of rule-based conversational agent in health care.

The use of CAs in health care is a recent occurrence that often translates to poor reporting of the CA development and evaluation processes, which may hinder the reliability of the research findings. To offer a systematic and transparent approach to CA development and evaluation, we previously developed and published a novel conceptual framework for designing, developing, evaluating, and implementing a smartphone-delivered, rule-based conversational agent (DISCOVER) [7] (Figure 1). The framework offers a comprehensive yet simple guide for the development of rule-based CAs that aims to address all the different facets of the development of such complex interventions. The framework was developed using the methodology by Jabareen [19] and was informed by a scoping review of rule-based CAs in health care [1], a narrative review of conceptual frameworks for the development of mobile health interventions [7], and our experience of developing a rule-based CA prototype to support healthy lifestyle changes to prevent type 2 diabetes [20,21]. The development of the DISCOVER conceptual framework was described by Dhinagaran et al [7].

The framework consists of 3 iterative stages of the CA’s design, development, and evaluation and implementation, complemented by 2 cross-cutting themes (user-centered design and data privacy and security). After development, to ensure the comprehensiveness and robustness of this framework, we validated the conceptual framework through consultation with experts in the nascent field of CA in health care [22].

### Aims of the Study

We aimed to present an updated conceptual framework for developing and evaluating CAs in health care, and to suggest a revised definition and classification of health care CAs. To this end, we performed in-depth, semistructured interviews with multidisciplinary experts in CAs for health care to evaluate and validate the conceptual framework for the design, evaluation, and development and implementation of rule-based CAs.

## Methods

### Overview

One-on-one semistructured interviews were conducted with international experts via Zoom (Zoom Video Communications) [23]. Prospective participants were invited if they had published ≥1 peer-reviewed paper on CA interventions. Purposive sampling was used to recruit participants, who were identified through a literature search of articles and reviews on mobile health, digital health, or CA interventions and consultation with a CA expert from our team. Snowball sampling was also used to recruit additional participants. In addition, study participants were asked to provide peer recommendations for further interviews. Email invitations were sent to 50 authors from diverse fields, such as computer science, health care, and digital health, to recruit between 10 and 20 participants, as it is common practice in qualitative interviews [24] and conceptual framework validation research [25,26]. After a positive response, follow-up emails were exchanged to fix an interview date and share further information about the study. Three days before the interview, we sent participants the informed consent form, a demographics survey, a voiceover PowerPoint presentation summarizing the conceptual framework to be discussed during the interview, and a link to join the videoconference meeting. Inclusion in the study was limited to participants who could communicate in English.

The interviews were conducted by 1 researcher (LM or AIJ) with the assistance of a second researcher (XL). LM is a pediatrician currently working full time in academic digital health research, whereas AIJ and XL hold psychology degrees with varied experience in digital health research that includes the design and development of CAs. The interviews were conducted using an interview guide with a series of illustrative questions (Multimedia Appendix 1) while acknowledging that participants’ responses may add questions not previously planned. The interview guide was developed by the research team and was piloted before the start of the study. The interview was divided into 2 sections. The first section enquired about the participant’s background; current role; experience with CAs; and an overview of the CA field, including the definition and classification of CAs and the advantages or disadvantages of using them in health care settings. The second section explored participants’ views on the framework’s overall design and individual components. The interview questions addressed the clarity and completeness of the framework, any unnecessary or missing steps, potential rearrangement of the current framework flow, and the rationale to justify each opinion. Interviews were conducted via Zoom between June and September 2022 and lasted between 60 and 90 minutes. Participants were interviewed once, except for 1 interview that was completed in a second meeting. The interviews were audio recorded and complemented with the researcher’s notes on the conversational aspects that could not be captured in the audio recording.

### Ethical Considerations

This study was approved by the Institutional Review Board of Nanyang Technological University (IRB-2021-816). Informed consent was obtained from all participants before the start of the interview. Participants received a US $20 web-based retailer voucher as compensation.

### Data Analysis

All interviews were transcribed verbatim by LM and XL using proprietary transcription software from the Lee Kong Chian School of Medicine Digital Learning team [27] and Microsoft Word automated transcription service [28]. Transcripts were checked for accuracy by the researchers and analyzed using thematic content analysis described by Burnard [29]. The transcribed data were analyzed sequentially, and inductive coding was used to generate the common themes and subthemes. Interview data were analyzed independently and in parallel using NVivo 12 software [30] by LM and XL. The researchers met regularly to discuss the interview coding, and disagreements were resolved through discussion and consensus. The report followed the Standards for Reporting Qualitative Research checklist. The definition and classification of CAs were supplemented by the authors’ previous and ongoing work, consisting of a series of scoping reviews on several aspects of health care CAs [1,7,31] and an analysis of definitions presented in several reviews [3,4,32-40] and a book [5].

## Results

### Overview

A total of 12 experts were interviewed. Most participants (9/12, 75%) had previous experience in developing rule-based CAs, whereas 25% (3/12) of the participants had developed hybrid CAs that combined rule-based decision trees with natural language processing. Table 1 presents the participants’ demographic information.

The findings from the expert interviews were organized into 2 distinct sections. Multimedia Appendix 2 presents participants’ quotes on the several topics discussed in the interviews.

**Table 1:** Participants’ demographic information (N=12).

Characteristics			Values
**Position, n (%)**			
	Professor	4 (33)	
	Associate or assistant professor	2 (17)	
	RF^a^ or senior RF	3 (25)	
	Research associate or doctoral student	3 (25)	
**Workplace, n (%)**			
	Academic institution	9 (75)	
	Research institute	3 (25)	
**Country, n (%)**			
	Australia	2 (17)	
	China	1 (8)	
	Taiwan	1 (8)	
	Singapore	2 (17)	
	Switzerland	4 (33)	
	United States	2 (17)	
**Field of expertise^b^, n (%)**			
	Computer science or artificial intelligence	2 (25)	
	Medical informatics, digital health, or digital mental health	8 (67)	
	Medicine (family medicine)	1 (8)	
	Psychology	2 (17)	
	Marketing, consumer behavior, and HCI^c^	1 (8)	
**Years of experience, n (%)**			
	<5	6 (50)	
	5-10	3 (25)	
	>10	3 (25)	
**Age (years), n (%)**			
	21-30	1 (8)	
	31-40	4 (33)	
	41-50	5 (41)	
	>60	2 (17)	
Number of publications on CAs^d^, median (range)			4.5 (2-150)
Number of publications on CAs, mean (SD)			19.6 (41.9)

^a^RF: research fellow.

^b^This section does not add up to 12, as 1 participant reported >1 field of expertise.

^c^HCI: human-computer interaction.

^d^CA: conversational agent.

### Defining and Classifying CAs

All participants defined CAs as computer systems that use natural language to interact with users. In general, this definition was broad and encompassing and included voice assistants such as Siri or Alexa, which usually engage briefly with users in a “transactional” (P003) manner. At the same time, CAs often “have some kind of coherent discourse” (P003). Although some participants considered terms such as *chatbot* and *conversational agent* synonyms and used them interchangeably, most participants distinguished between them. In general, CA was regarded as a “bit broader” (P002) term that encapsulated different types of agents, including chatbots. Chatbots were seen as referring specifically to “text-based and rule-based conversational agents” (P001). However, 1 participant distinguished CAs from chatbots and other types of dialogue systems, as CAs could engage in an empathic, personalized conversation with the user and “develop relationships” (P009) that emphasized the “social and emotional aspects of the interaction in addition to the health care tasks” (P003).

Experts classified CAs according to 13 different categories. These included commonly used ones such as CA *input modalities* (text based or speech based), *purpose* (domain specific or general purpose), or *response generation method* (rule based, artificial intelligence [AI] based, or hybrid). They also proposed other categories; for example, CAs were classified according to the *development modality* as “bespoke or off-the-shelf conversational agents” (P001) or according to the *deployment platform* as app based, often as a stand-alone CA or integrated into a website or another platform such as Facebook Messenger, Telegram, or Slack; according to the “type of communication, is it supportive, or is it just information providing” (P006). In addition, participants also categorized CAs according to their *appearance* (disembodied, embodied, or social robots), *length of interaction* (short term, medium term, and long term), *personality* (coach, peer, or expert), *type of CA-user interaction* (transactional or relational), the *inclusion of human support*, “where in the patient journey it’s used” (triage, appointment management, medication adherence, others; P007), or the *domain* (health care, commerce, business, or others).

CAs in health care settings were seen as “a scalable way of delivering...personalized health services to people” (P010). Participants noted the advantages and disadvantages of using both rule-based and AI-based systems. There was widespread consensus among experts that, at present, rule-based CAs should be the norm in health care. Rule-based CAs require developers to predefine all dialogue turns, which allows greater control of the conversation flow, constituting their main advantage in health care settings. However, there are several disadvantages associated with rule-based CAs. For example, rule-based systems lack flexibility and may not address all user concerns. Moreover, CA interventions that require ongoing interactions with a user may lead to disengagement and boredom, as responses will be similar each time. Alternatively, AI-based CAs were seen as offering attractive, flexible, and innovative systems that may increase long-term user engagement. However, experts considered that current AI technology does not ensure safe and reliable conversations, particularly in health care settings. Finally, experts acknowledged that CA development is a complex, labor-intensive task, which applies to rule-based CAs and AI CAs.

### A Conceptual Framework to Design, Develop, and Evaluate and Implement CAs

#### The Role of a Conceptual Framework for the Development of CAs in Health Care

Participants felt a conceptual framework to guide the development of rule-based CAs was useful. It provides researchers and developers with a guideline for CA development and “a benchmark for analysing it” (P007). Notably, a conceptual framework is helpful for “writing research proposals” (P003) or to “consult it to make sure I wasn’t missing something” (P010). Furthermore, the framework was helpful for “developers or for companies” as they may “get caught up in the business model” and “[may not] draw from conceptual or theoretical frameworks as much as they should” (P005).

All participants agreed that the framework assessed in this study could be adapted for developing AI-based CAs, as many considerations also apply to AI-based CAs. An exception was the development of the conversation flow because AI-based CAs require the use of training data sets rather than decision trees. In addition, experts pointed out that AI-based CAs’ training data should be representative of the target population, that researchers should be able to explain the algorithm’s evolution, and that the CA responses should be accurate and not compromise users’ safety.

Experts valued the content and layout of the existing conceptual framework. The visual presentation was considered “simple to understand and not overly complicated” (P008). Participants agreed to use a circular diagram as it represented the iterative nature of the CA development cycle. They also suggested the use of “non-standard fonts” (P008), “some more colors” (P006) to differentiate the stages, “emphasizing the arrows” (P008) to highlight the framework’s iterative nature, and modifying the placement of the cross-cutting themes to avoid linking them to the specific stages of the framework. However, they suggested adding more details to each stage to make the diagram more self-explanatory. Participants had varied views about the *framework name*, appreciating that it was simple and easy to remember, although they might “not directly connect it with the framework for chatbot development” (P002).

#### Design of CAs

Participants agreed with the following existing concepts: defining the CA goal, determining the CA identity, identifying target users, selecting the delivery interface, and assembling a multidisciplinary team. They also added a new concept: specifying the evaluation outcomes (Multimedia Appendix 2). *Defining the CA goal* was highly relevant. Participants expressed that a clear and in-depth understanding of the health care problem is essential to “define the goal based on the problem” (P001). Still, they highlighted the relevance of defining “the central aim, why are we doing this?” (P005). Research methods used for needs assessment, such as reviews, focus group discussions, interviews, and surveys, play an important role not only in defining the goal but “for all of the content to make sure it’s understandable and acceptable by [the] end audience” (P003).

The *identity of the CA* is important “in terms of who people trust and don’t trust and the degree of which they think the information they’re getting is trustworthy” (P009). One fundamental aspect of defining the CA identity design is to adapt the content to the local culture, including the local nuances that may make the agent more relatable to the target audience.

The *target users* refer not only to the end user of the intervention but also to the broader social circle formed by family, other members of the user’s social network, and health care providers. This is particularly important if the CA intervention will be used in the health care system or if the target population includes older adults or individuals with intensive care needs. Developers may also consider the setting where the intervention would occur.

*Selecting the delivery interface* is “a key choice” (P012). Researchers “need to realize that there are differences with the platform” (P010), in terms of users’ acceptance of the different tools, and regarding the technical “capabilities of the delivery interface” (P008). The choice of platform may even determine the type of outcome measures to be collected during the CA evaluation.

Assembling a *multidisciplinary team* was considered essential when designing a CA-based intervention. The team may include a “linguistic professional” (P012) to ensure that the text will be adequate for the end user, content participants familiar with traditional face-to-face interventions, and digital health intervention specialists. The team composition may also depend on the nature of the research collaboration, as 1 expert pointed out, “if I'm working with [an industry stakeholder], they tell me the team” (P008). Finally, participants suggested that it is also important “to define the roles of everyone within the team because sometimes that’s a bit unclear” (P007).

Experts noted that the *type of outcomes* to be measured and the time points at which these measurements may occur during the evaluation stage should be defined during the design stage. It is crucial to consider the sources of “the data that you’re going to use...it’s participant reported and sensor data, but also electronic medical record data, health systems data, staff data, leadership data, focus group data. So, it’s broader than just participant data” (P009). Planning for sensor data collection is essential as it must be coded while developing the CA. Special care should be taken to ensure the correct spelling of data variables, as minor orthographic mistakes may render the data unavailable.

#### Development of CAs

Although participants agreed on the concepts included in this stage, they suggested adding additional details to the diagram to make the content more self-explanatory, particularly for inexperienced developers.

The length of the dialogues was repeatedly mentioned as a critical consideration to be discussed early in the development process. It may take different interpretations, referring to the length of each CA turn (how many lines of speech), the length of the dialogue (number of times each speaker has a turn), and the total number of sessions required to deliver the intervention. The dialogues may convey an empathic, nonjudgmental tone, as this may influence user engagement and bonding with the CA. Moreover, it is essential to consider the language that best translates the content of face-to-face interventions into the constrained length of CA dialogues.

Participants agreed that all content in the conversations should be *evidence based*, referring to the adherence to current best practices as stated in the scientific literature and clinical guidelines and the dialogue structure that should emulate “seasoned clinicians” (P009). Health care CAs must also have adequate systems to respond to possible *medical or psychological emergencies*. For example, they may provide crisis helpline phone numbers, include advice for the initial management of common symptoms, or hand over emergency management to a health care provider.

*Error management* includes inadvertent adverse events and mistakes (either from the CA or a person who enters the wrong data). However, adverse events are rarely reported in CA studies, which increases the importance of collecting “information if a person deteriorates in an outcome” (P006). Moreover, 1 expert highlighted that adverse event management might be challenging if users associate the CA with their health care provider and assume that the agent would identify adverse events when they occur. Therefore, developers may include safeguards to assist users adequately if an adverse event occurs.

*Personalization* implies that the “chatbot can maybe store the user preferences during the conversation” (P011). Personalization is essential, and it is considered a hallmark of CAs, which may draw information from several sources to “tailor the dialogue specifically to [the user]” (P009).

Finally, when defining the *platform,* researchers should consider the size and expertise of the multidisciplinary team. Although large teams may develop the CA “from scratch” (P001), smaller teams with limited coding expertise may “use some platforms, [such as] the Alexa skills or Juji” (P001).

#### Evaluation and Implementation of CAs

Participants agreed with existing evaluation and implementation topics, such as usability assessment, user engagement, intervention efficacy, and effectiveness, and suggested new topics, such as technical evaluation. They also pointed out that CA evaluation is an iterative process that often starts during content development to ensure that conversational turns occur as planned and are adequate to fulfill the CA aim. In addition, during the evaluation process, researchers should consider how the test results can be used to improve the CA further.

Participants offered varying definitions of what *usability* entails. For example, usability was defined as “not just A/B testing, but qualitative studies of what users think about the agent” (P003) and referred to user experience as a dimension of usability. Concurrently, it is important to use standardized tools to measure usability, such as the System Usability Scale [41] or the Chatbot Usability Scale [42], as “researchers tend to develop their own usability test” (P002), and this may limit comparability. *User engagement* is another important consideration, particularly in longitudinal interventions.

The *technical evaluation* of CAs has not been discussed in the original framework. However, such evaluation is important to ensure that the CA is ready and functional before evaluating it in clinical trials, probably during development. It includes not only crashes and bugs but also the evaluation of the CA content credibility and data privacy and security safeguards, among others.

In general, participants agreed that, although infrequently mentioned, *economic evaluation* of the end product is essential to ensure the viability of the CA beyond clinical trials and consider the cost-effectiveness of the intervention. Moreover, expert P002 suggested that economic evaluations could be considered in all 3 stages of the CA development process, whereas expert P003 argued that “economic evaluation is rarely done” in research settings.

Finally, 1 expert suggested modifying the framework to “having four boxes and four quadrants” as *implementation* is different from evaluation and requires “different skill sets altogether” (P009).

#### Cross-Cutting Themes

Participants agreed with the existing cross-cutting themes of user-centered design and data privacy and security and suggested 2 additional ones: ethics and long-term sustainability.

Including *user-centered design* was an important aspect of CA design and evaluation. However, participants clarified that user-centered design is the name of a specific design process characterized by the user’s influence in the design and should not be used to conceptualize the importance of user involvement in the design process. Participants distinguished between being user centric; seeking end users’ opinions through surveys, interviews, or other qualitative methods during the CA development; and directly involving users in the design and development of the CA, as seen in participatory design frameworks. However, users may express divergent views, which may be difficult to reconcile when deciding the features to be included in the intervention.

The inclusion of data *privacy and security* was welcomed by all participants, who agreed that this topic is critical and sometimes not adequately discussed in the scientific literature. Data privacy and security were perceived as complex issues with multiple connotations, including users’ behavior, which could be “very paradoxical” (P008), meaning that users may assert themselves as genuinely concerned about privacy but act as if it is not important at all. Users’ concerns about data management may correlate with the extent of personal data collection. They may require more detailed explanations of how researchers will use their data if the app collects large amounts of personal data. In addition, researchers need to find a “balance between getting as much data as possible... [to] make the intervention more personalized, but also [not] to cross the boundaries of what people feel comfortable with sharing” (P007). Participants also emphasized the importance of developing adequate data management plans that align with their countries’ current data protection laws during the design stage. Finally, future handling of research data may be challenging, as technology development may facilitate the reidentification of initially anonymous data.

*Ethical considerations* were viewed as an essential cross-cutting theme from 2 different perspectives. One expert referred to the overarching ethical principles that should guide all health care interventions, “principles like nonmaleficence, beneficence, autonomy, fidelity” (P005). In contrast, another expert mentioned that when applying for ethics approval, researchers may need to modify the project execution to suit the current ethical best practices.

Finally, expert P010 suggested assessing early in the development (and as an ongoing theme) “where [the intervention] fits in the bigger health system” to ensure that researchers develop a sustainable, cost-effective system that addresses a real and required health care need.

## Discussion

### Principal Findings

This study presented the views of 12 multidisciplinary CA experts on the definition, classification, and development and evaluation of CAs in health care. Experts generally distinguished CA as an overarching term that contains several types of agents, including chatbots, and they proposed 13 categories to classify CAs. Participants agreed with the overall conceptual framework for designing, developing, evaluating, and implementing health care CAs and offered suggestions to improve the framework. Experts also agreed that the framework could be adapted for the development of AI-based CAs if the technology can ensure their safety and reliability in health care settings.

**Figure 2:**
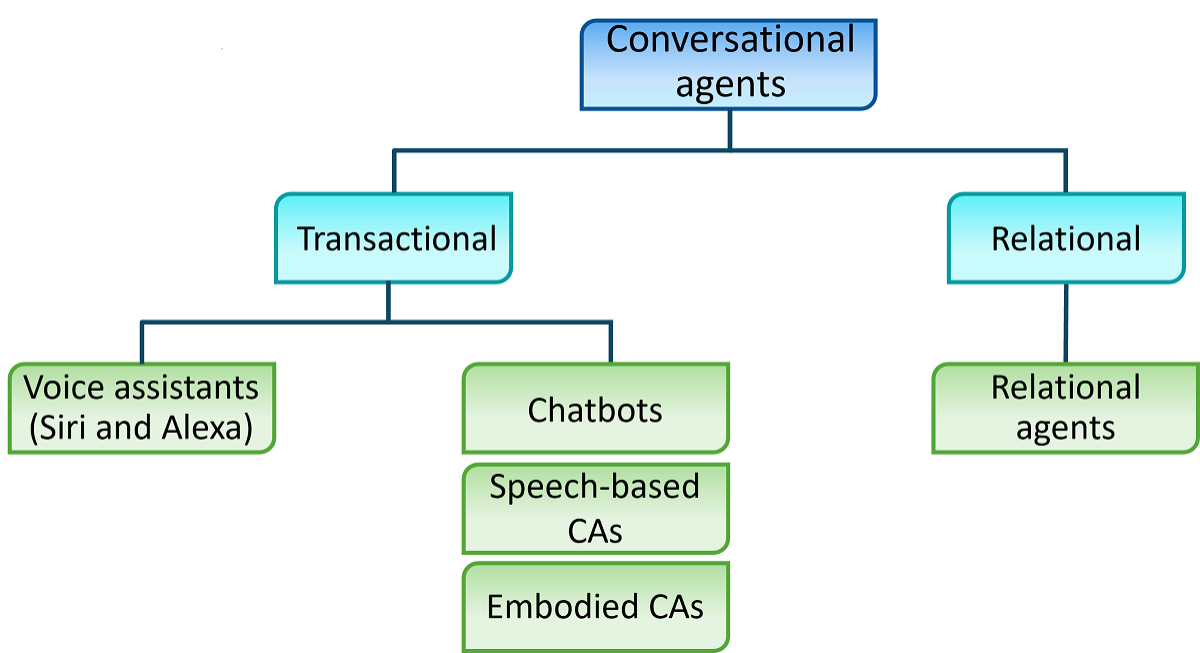
Types of CAs according to their characteristics and functions. CA: conversational agent.

Although participants offered diverse definitions, they most clearly defined CA as an encompassing term that includes all subtypes of conversational interfaces. Thus, based on the experts’ descriptions of CA and our research, we propose the following definition of CA: CAs are digital interfaces that use natural language to engage in a synchronous dialogue using ≥1 communication modality, such as text, voice, images, or video. This definition includes a variety of CAs, such as transactional, single-turn voice (or virtual) assistants (eg, Siri or Alexa); text-based and often rule-based CAs or chatbots; and complex, embodied CAs able to engage in verbal and nonverbal communication with users. In addition, a subset of embodied agents, referred to as relational agents, aims to “build and maintain long-term, social-emotional relationships with their users” [43], a feature that sets them apart from other CAs (Figure 2).

**Figure 3:**
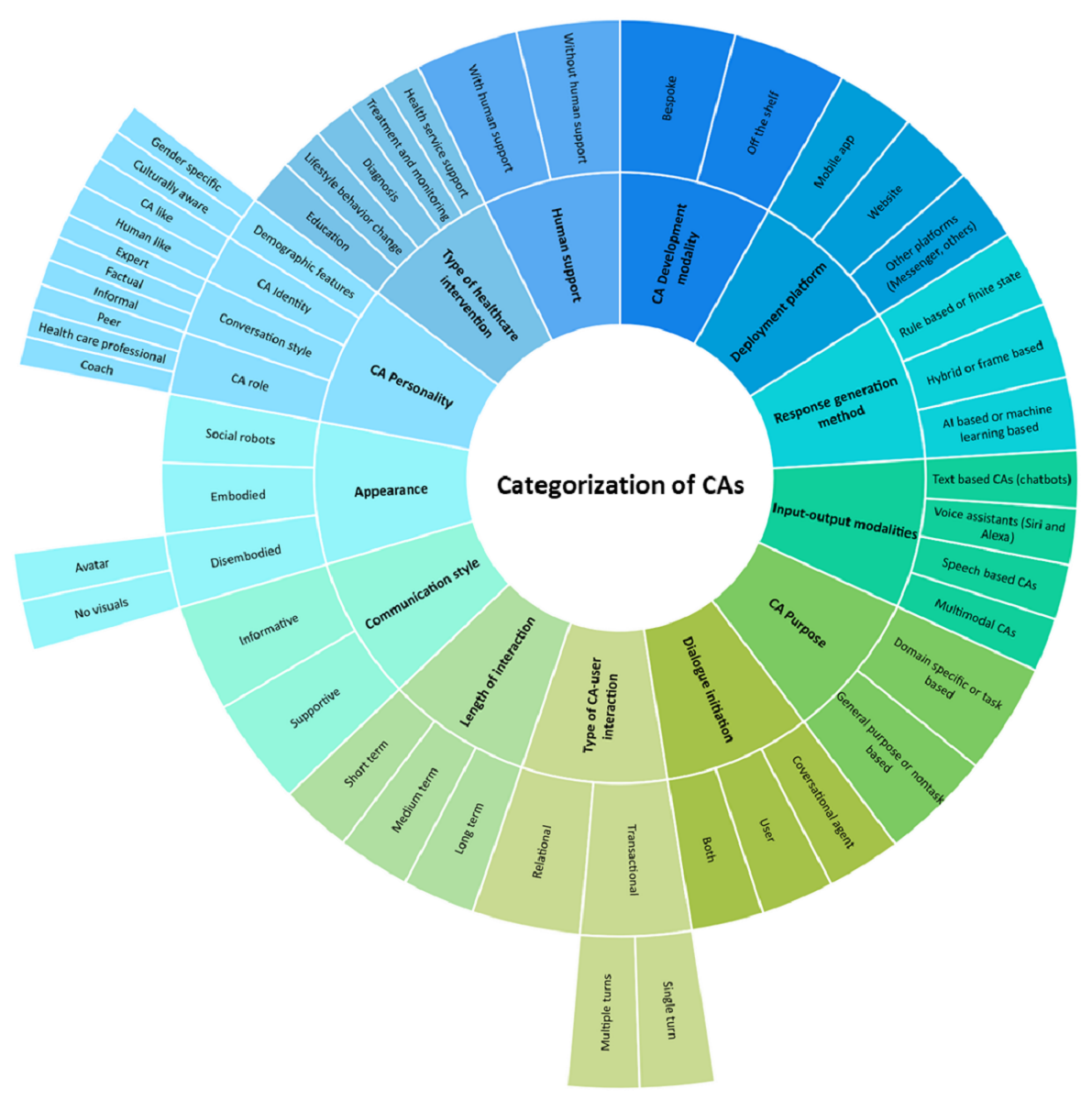
Experts’ categorization of CAs. CA: conversational agent.

Participants suggested 13 ways to categorize CAs: domain, input modalities, purpose, response generation method, development modality, deployment platform, communication style, CA appearance, length of interaction, CA personality, type of CA-user interaction, the inclusion of human support, and where in the patient journey it is used. We compiled these categories; added a category, dialogue initiation, from the systematic review by Laranjo et al [18]; and removed the domain category as it was not specific to health care. We also expanded the CA personality category and merged the location in the patient journey to create the “type of health care intervention” category with additional information from our previous work [1]. The result was a novel classification of health care CAs, consisting of 13 categories describing the CA appearance, communication modalities, and uses in health care settings. Denecke and May [44] recently published a technical-oriented taxonomy for health care CAs aimed at improving the reporting of the technical aspects of CA development. The authors included 18 categories grouped into 4 dimensions (agent appearance, setting, interaction, and data processing). The taxonomy included 8 categories that overlapped with our classification, including CA personality, appearance, length of interaction, response generation method, purpose, human support, input-output methods, and deployment modalities. The CA categorization is shown in Figure 3.

Experts’ recommendations largely validated the content and structure of the previous version of the conceptual framework [7], suggesting that most elements of the original framework are congruent with participants’ knowledge and experience in developing health care CAs [7]. The framework was also aligned with other frameworks guiding the design and development of digital health interventions in general [45,46]. Participants also provided valuable suggestions to improve the framework’s look, content, and structure, including adding more information to the diagram to make it more self-explanatory and performing technical evaluations of the system early in the development cycle to ensure the viability of the prototype before starting costly, patient-facing tests. Furthermore, participants suggested the inclusion of ethics as a cross-cutting theme. Experts shared that the biomedical ethics principles of autonomy, nonmaleficence, beneficence, and justice [47] should guide the design of digital health care interventions. Of particular importance are the unequal access to technology associated with inadequate digital literacy or economic disadvantage, data privacy and security breaches, and potential risks of bias and harm [47-49]. Adequate measures to reduce these risks should be considered and implemented in all stages of the CA development process.

Our conceptual framework focuses on the development of rule-based CAs. However, experts agreed that its principles could be adapted to AI-based CAs if additional guidance on topics specific to creating AI algorithms is added in the framework’s development stage. Nonetheless, participants were cautious about using AI-based CAs in health care settings, given the associated risk of misunderstanding posed by systems that are unable to contextualize the conversation or understand the nuances of words and metaphors that often convey a meaning different from the textual discourse. However, the field of conversational AI has seen significant developments over the last year, particularly with the public release of OpenAI’s ChatGPT [50] in November 2022, which is a large language model that uses complex algorithms and reinforcement learning with human supervision to generate a coherent output. ChatGPT has recently been credited with passing the USMLE (United States Medical Licensing Exam), a standardized set of 3 exams required to obtain medical licensure in the United States [51]. These developments may increase the interest in adopting AI in health care settings. However, health care providers and researchers should be aware of the limitations of large language models to provide reliable and accurate responses [52], as well as issues of bias in the data [53], user safety, algorithm transparency, explainability, and liability [54-56], which are essential for ensuring the safe and reliable provision of health care.

### Conceptual Framework for Health Care Conversational Agents

**Figure 4:**
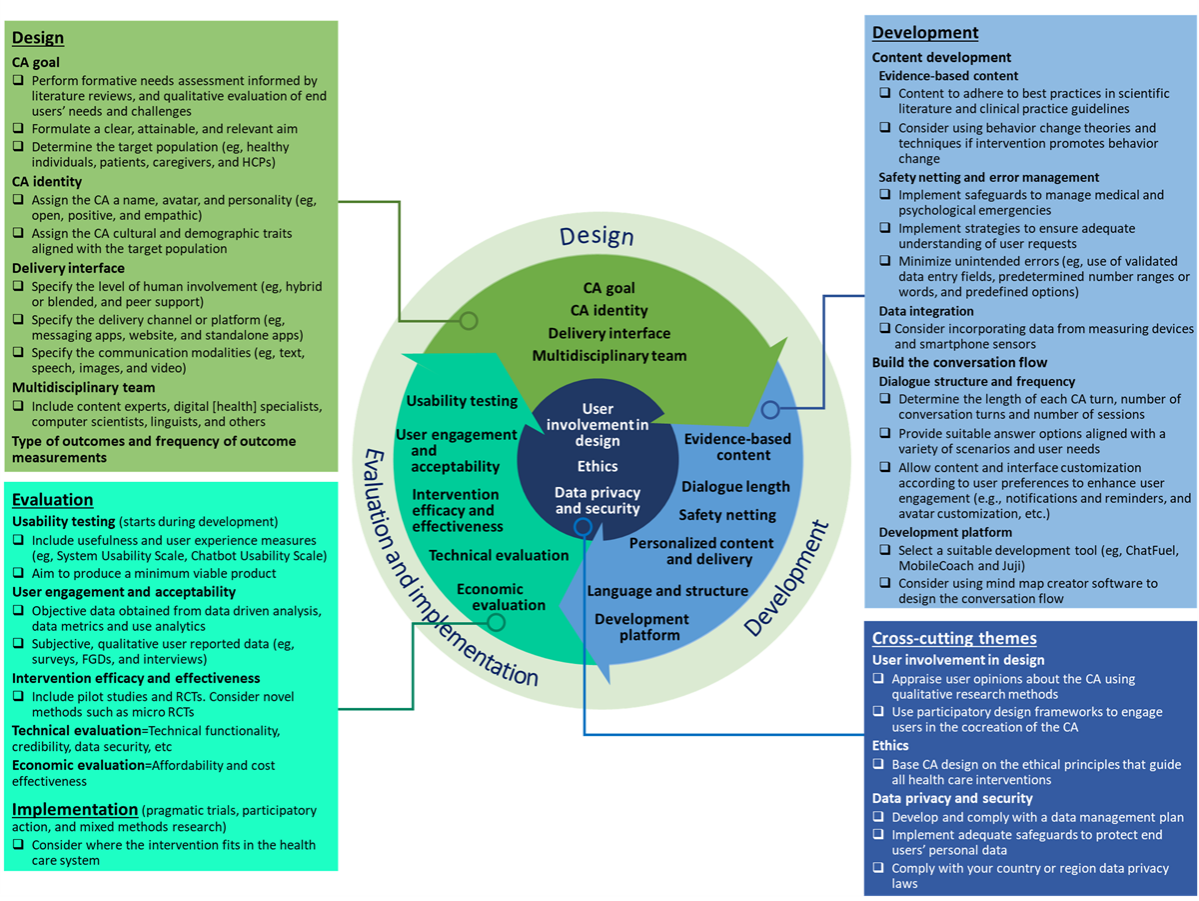
Conceptual framework for healthcare conversational agents (CHAT). CA: conversational agent; HCP: health care provider; FGD: focus group discussion; RCT: randomized controlled trial.

Participants’ inputs and suggestions were adopted to develop an improved version of the conceptual framework, renamed conceptual framework for health care conversational agents (CHAT). This updated version incorporates improvements to the visual presentation and content of the framework. We also renamed the framework in response to expert comments that the previous name was not easily relatable to CAs. Visually, the structure was modified by moving the cross-cutting themes to the center to avoid linking specific cross-cutting themes to particular stages of the framework. We also emphasized the arrows illustrating the iterative process of CA development, including the term *conversational agent* within the framework, and standardizing each stage’s description. Finally, following suggestions to make the framework diagram more self-explanatory, we added the development stage, and we created a checklist to supplement the information presented in Figure 4.

CHAT consists of 15 key topics grouped into 3 stages: the first stage, *design*, includes determining the CA goal and CA identity, selecting a delivery interface, and assembling a multidisciplinary team. The second stage, *development*, includes 2 sections: developing the content highlights the use of reliable, evidence-based sources; incorporating error management and safeguards to avoid user harm; and considering data integration with phone sensors or external devices. Building the conversation flow, considers the dialogue length, language and structure, personalization, and the CA development platform. The third stage, *evaluation and implementation* of the CA, includes the assessment of usability, user engagement, intervention efficacy and effectiveness, as well as technical and economic evaluations. Finally, these stages are supplemented by 3 cross-cutting themes: user involvement in design, ethics, and privacy and security, which are relevant at all the framework stages.

The CHAT framework was developed to assist academic research teams of different sizes and resources in planning the design, development, and evaluation of rule-based CAs. However, the framework may also benefit developers or companies to ensure compliance with evidence-based principles.

### Strengths and Limitations

This study has several strengths. First, the interviews were conducted with experts from various fields, from computer science to medicine, who shared their unique experiences working with CAs. Second, we used a comprehensive interview guide that allowed for an in-depth evaluation of the conceptual framework and the broader CA domain.

However, this study had several limitations. First, most participants were digital health or medical informatics specialists, which may have biased the study results toward more technical aspects of CA design and development. Second, most study participants worked in academic settings. Therefore, the interviews may have overlooked specific elements related to the design and development of CAs for commercial for-profit companies. Third, an early version of this conceptual framework was used to guide the development of a healthy lifestyle CA to prevent type 2 diabetes. Further research should evaluate the use of the framework for the design of different types of CAs. Further assessment is required to evaluate the relevance of the framework steps in designing effective CA interventions. Finally, the adaptation of this framework for AI-based CAs will require more details, particularly in the development stage of the framework.

### Conclusions

The use of CAs, which are complex and diverse digital health interventions in health care settings, is increasing. We invited CA experts to define, classify, and discuss the steps required to develop CAs in health care settings. According to experts, CAs are digital interfaces that use natural language to engage in a synchronous dialogue using ≥1 communication modalities such as text, voice, images, or video. CAs can be classified into 13 categories: response generation method, input and output modalities, CA purpose, deployment platform, CA development modality, appearance, length of interaction, type of CA-user interaction, dialogue initiation, communication style, CA personality, human support, and type of health care intervention. Finally, the CA development process is presented as CHAT, which consists of 3 stages of design, development, and evaluation and implementation of CAs, complemented by 3 cross-cutting themes: user involvement, data privacy and security, and ethics.

## Appendix

Multimedia Appendix 1 Semistructured interviews guide.

Multimedia Appendix 2 Selected experts’ quotes that illustrate the study findings.

## Abbreviations

AI: artificial intelligence
CA: conversational agent
CHAT: conceptual framework for health care conversational agents
DISCOVER: designing, developing, evaluating, and implementing a smartphone-delivered, rule-based conversational agent
USMLE: United States Medical Licensing Exam
