# Supplementary material for: Conversational Agents in Health Care: Expert Interviews to Inform the Definition, Classification, and Conceptual Framework
Source: J Med Internet Res. 2023 Nov 1;25:e50767. doi: 10.2196/50767 (PMC10652195; doi:10.2196/50767)
Supplement: Multimedia Appendix 1 [file jmir_v25i1e50767_app1.docx]

**Multimedia Appendix 1**: Semi-structured interviews guide

**Introductory statement**

Thank you very much for agreeing to participate in this interview. May I first check if you had the chance to sign the informed consent form and the “Participant Information Survey”? (Yes – thank you very much / No – no worries, we are sharing the web link through the chat function. Alternatively, you may want to scan the QR code shared now on the screen)

We would like to ascertain your views on a new conceptual framework developed by our team, aimed to assist developers, researchers, or other interested parties in the design, development, and implementation of rule-based conversational agents. A summary of the conceptual framework was sent to you a few days ago as a voice-over presentation. Did you have the chance to watch it? (Yes – thank you / No – no worries, you will be able to see it after this introduction)

The interview will last about one hour, but no longer than 90 minutes. It will be audio and video recorded, but only the audio recording will be saved for transcription. Your views will remain anonymous in the transcription, analysis, and publication of the research results. All the information collected, including the recordings, will be stored in the university’s secure servers for 10 years, in accordance with research integrity guidelines.

Do you agree for the interview to be recorded?

- PRESENTATION -

We will divide the interview into two sections: in the first section we will ask you some questions about conversational agents in general, and in the second section we will discuss the DISCOVER conceptual framework, we just described in the presentation. But before we start, kindly complete the survey we just shared through the chat function.

**Experts’ previous experience**

1. Could you please introduce yourself?
   1. **Probe if not mentioned earlier**: Could you please tell us how you came to be involved in conversational agent research?
2. Can you share with us your experience in the development of conversational agents?
   1. How many CAs have you and your team developed?
   2. What type of CA have you developed?

**Prompts**: Rule-based/AI/mixed

ECA/non-ECA/both

Text/speech/other

1. How did your previous work inform the development of your conversational agent?
2. Can you describe the principles or framework used for the development of the conversational agent(s)?

**Conversational agents**

1. Conversational agents’ definition and classification.
   1. How do you define “conversational agent”?
   2. Are there any other terms used to define “conversational agents”?
   3. What is, in your opinion, the “correct” term?
   4. Do you consider all these terms synonyms?

**Probe:** If no, what makes these terms different?

**Probe**: Does the term used to define the conversational agent/ chatbot differ according to the input method? (text-based, speech-based)?

- 1. Do you consider platforms such as Siri or Alexa a conversational agent/ chatbot? Should they be considered the same as a text-based conversational agent (Wysa for example)
  2. How would you classify conversational agents?
  3. What, in your opinion, are the advantages and disadvantages of rule-based conversational agents?
  4. What about the advantages and disadvantages of AI-based conversational agents?

**Probe:** What is your opinion on rule-based vs AI-based conversational agents in healthcare? You mentioned earlier that you were involved in the development of [rule-based/AI] CAs. What made you choose [rule-based/AI] as the basis of your CA?

1. Development of a rule-based conversational agent
   1. What are, in your opinion, the main steps to developing a rule-based conversational agent?
      1. Which steps did you consider the most challenging, and how did you overcome those challenges?
      2. If you could go back in time, what would you do differently in the development of your CA?

**Probe**: Why (would you want to do it differently)?

- 1. In your opinion, how would the CA development team be integrated?

**Conceptual framework**

1. Thinking of the conceptual framework diagram we shared with you earlier (this week),
2. What’s your opinion about having a conceptual framework for the development of rule-based conversational agents?
3. What are your views on the name of the framework?
4. What are your views on the overall design?
5. What are your views on the visual presentation of the framework?
6. What are the most helpful characteristics?
7. What aspects of the framework would you improve?
8. Are there any aspects of the framework that you think are redundant?
9. Are there any aspects of the framework that you think are missing?
10. Now I will ask you a few questions about the specific sections of the framework:
11. As a CA developer, are the topics covered in the Design stage account for all the aspects that should be considered during the initial stages of a CA development process?
12. Is there anything missing?
13. Would you add anything to the framework?

**Probes:**

- 1. Are there any other aspects of “Defining the goal” we should take note of?
  2. Are there any other aspects of “Creating an identity” we should take note of?
  3. Assembling the team
  4. Selecting the delivery interface

1. Moving to the “Development” stage, are the topics covered here, regarding content development and building the conversation flow, relevant and sufficient in this second stage?
2. Is there anything missing?
3. Would you add anything?

**Probes:**

1. Are there any other aspects of “Developing the content” we should take note of?
2. Are there any other aspects of “Building the conversation flow” we should take note of?
3. Following now with the “Evaluation and Implementation” stage, would you say that the topics included are adequate for this third stage?
4. Is there anything missing?
5. Would you add anything?

**Probes:**

- - 1. Are there any other aspects of “Usability testing” we should take note of?
    2. What about “Pilot studies and RCTs”? we should take note of?
    3. And “User engagement and acceptability” we should take note of?
    4. Are there any other aspects of “Economic evaluation” we should take note of?
    5. Are there any other aspects of “Implementation” we should take note of?

1. Finally, moving to the cross-cutting themes, what are your views about the inclusion of user-centered design here?
2. What about data privacy and security?

**Probes:**

1. Are there any other aspects of “User-centered design” we should take note of?
2. Are there any other aspects of “Data privacy and security” we should take note of?
3. Would you add any other topics here?
4. Or remove them?
5. Finally, we would like to hear your views about the possibility to adapt this framework for AI conversational agents as well
6. Would the framework in the current form be adequate to guide the design of AI CAs?
7. If not, what aspects of the framework need to be modified in order to guide the development of AI CAs?
8. What elements are missing? What should be added to the framework?
9. What elements, if any, are redundant?
10. Given the chance, would you want to use this framework for your previous works?

We have come to the end of the interview. Is there anything not covered but you would like to add?

If you have any more comments or questions after this interview, please feel free to contact us via email for more information

If the participant asks for further information/paper about the framework can share the following doi (JMIR preprint): [10.2196/preprints.38740](https://preprints.jmir.org/preprint/38740)

Thank you for your time and effort in participating in this interview. We will shortly send you an Amazon gift card to express our gratitude for participating in this interview.
