# Supplementary material for: Conversational Agents in Health Care: Expert Interviews to Inform the Definition, Classification, and Conceptual Framework
Source: J Med Internet Res. 2023 Nov 1;25:e50767. doi: 10.2196/50767 (PMC10652195; doi:10.2196/50767)
Supplement: Multimedia Appendix 2 [file jmir_v25i1e50767_app2.docx]

**Multimedia Appendix 2**: Selected experts’ quotes that illustrate the study findings

| **Themes** | | | **Illustrative Quotes** | **Expert** |
| --- | --- | --- | --- | --- |
| **Defining and classifying conversational agents** | | | | |
|  |  | CA definition | “*Conversational agents are dialogue systems where users are communicating with the system using natural language.”* | P002 |
|  |  |  | “*a system that mimics human interaction., either rule-based or artificial intelligence-based.*” | P006 |
|  |  | CA classification | “…*main classification is based on modalities like text, voice, you know, sometimes embodied conversational agents. There is also task-based, non-task based … a bespoke or off-the-shelf conversational agent ... task-based, non-task based, domain-specific, general purpose*…” | P001 |
|  |  | Rule-based CAs the norm in healthcare | “…*rule-based definitely, I think currently should be the norm in the healthcare area due to safety*” | P001 |
|  |  |  | “… *we have a set of defined rules that helps us as researchers or as developers to create systems that are reliable, so we know how they will react. This is good for health care because we need reliability to avoid patient harm*…” | P002 |
|  |  | Rule-based CAs lack flexibility | “*when* [the user] *has to use the system several times that* [the user] *gets bored, because* [the user] *knows what the system is asking* [his/her] *or what just answering to [his/her] questions*.” | P002 |
|  |  |  | “*the answer options that are given in a rule-based* [CA] *might not fully reflect something that the user might want to answer.*” | P007 |
|  |  | Advantages of AI-based CAs | “*if the conversational agent truly learns and truly improves itself in the conversation, it can cover a broader range of issues and cases and individual problems or things that the user might bring up*.” | P004 |
|  |  | AI technology requires further development | “*We don't yet have techniques for extended coherent discourse using machine learning, … if you're talking about machine learning-based understanding of natural language input by users for healthcare, there is no way to guarantee safety of those systems*.” | P003 |
|  |  |  | “…*there are some words that can be ambiguous, and the machine can’t understand this ambiguity…*” | P012 |
|  |  | Is AI safe for healthcare? | “*I think there is space, but there has to be a first question you always ask. Which is, what if they make a mistake? Any harm come from them making a mistake? And if the answer to that is yes, then they shouldn't be used*.” | P003 |
|  |  | CAs development is labor-intensive | “[developers] *have to hard code and manually enter every individual response*” | P001 |
|  |  |  | “*it will require a lot of training, data training, like, for example, the first time maybe it will not work.*” | P011 |
| **A conceptual framework to design, develop, evaluate, and implement CAs** | | | | |
|  | The role of a conceptual framework for the development of CAs in healthcare | A conceptual framework is useful | “*I think having a framework means planning and strategizing and thinking it through ... I think it makes good sense to have a conceptual framework.*” | P002 |
|  |  |  | “*I think it could be very helpful... When we have such a framework, then we can just do or work along these this framework and ensure, hopefully, to produce at the end useful conversational agents in healthcare*.” | P011 |
|  |  |  | “*this is really important because it can provide a guideline for the developer, what should be considered when they develop a chatbot.*” | P011 |
|  |  | Areas that may benefit from having a framework | “*one area where it would be useful is in writing project proposals. We often want to be able to point to … a formal framework.* “ | P003 |
|  |  |  | “*I think it's very useful ... particularly for students when they are doing smaller interventions.*” | P006 |
|  |  | Framework adaptation for AI-based CAs | “*developing the content is simply different in AI-based systems. So maybe we just have to create conversations that the system can learn off. Maybe what is even more important … is defining the goal or the purpose.*” | P002 |
|  |  |  | “*Yes, to a certain degree, especially in the design part, and also evaluation and implementation, of course. The development will be different, but design and evaluation could certainly be the basis*.” | P006 |
|  | Overall impression of the existing framework | The conceptual framework structure is clear | “*It clearly states the steps that should be conducted when developing a conversational agent*.” | P002 |
|  |  |  | “*I think the domains, the design, development evaluation, implementation are good. And then the most helpful part is the specific content within each of those domains. It is really the key to this, and that's helpful*.” | P009 |
|  |  | Visual presentation | “*I think the fact that it's like a loop is helpful because it emphasizes that it is an iterative process*.” | P007 |
|  |  |  | “*the most helpful* [including] *the items inside the big theme … like each item will help us quite understand the framework*.” | P011 |
|  | Design | Defining the CA goal | “*defining the product while having in mind that this product needs to fit into a conversation or like text messaging format, understanding the limitations of this … and figuring out how much we can do, how much can go into this chatbot, and what is not possible and cutting functionalities as well.*” | P004 |
|  |  | CA identity | “*data generally says that people from the same cultural group are more trustworthy than not.*” | P009 |
|  |  | Target users | “*be clear about the setting the system will be used in. So, is this the patient at home? or do we need some data transfer to the health care professional?*” | P002 |
|  |  |  | “*who are the target users, it reminds me of what is my inclusion criteria. And then, there's got to be an exclusion criteria as well, there could be some people who are close, but maybe this doesn't apply … who are our target users? and who are not our target users?*” | P005 |
|  |  |  | “… *the target users are the users, it's also family members often … So that users are not only the primary users but also the secondary users* … [and] *in the hospital system … it's critical for success that the staff like it and it's good in their workflow … And also, leadership, is it meeting the overall goals of the institution?*” | P009 |
|  |  | Delivery interface | “… *the different possibilities for how it's delivered, that was definitely a key choice we had to make … I think you need to realize that there are differences with the platform*.” | P010 |
|  |  | Multidisciplinary team | “*putting together the team of people who will work on it, but I think it also can be useful to define, like, the roles of everyone within the team*, [be]*cause sometimes that's a bit unclear.*” | P007 |
|  |  |  | “*the team members, that's sort of a general term, but I think specifically, so there's the users, there's the staff or the sort of the people external to the system and the user that impact its usage. And then, at a higher level, is the leadership. So, the team members include all those people.*” | P009 |
|  |  | Type of outcomes | “[Data is] *something to be defined or mentioned in the design or development stage already … define the data points you want to monitor and make sure that you have the tools to collect the dat*a.” | P004 |
|  | Development | Development stage needs more details | “*it's clear and well-structured, and that I have an overarching idea of the process, but it's still like very abstract without the voiceover.*” | P004 |
|  |  |  | “*in the development part, developing the content, building the conversational flow, there could be more detail into what steps do you go through when you want to develop, you know, the content or even building a conversational flow.*” | P007 |
|  |  | Designing the dialogues | “*labor-intensive situation, there is a lot to cover and very resource intensive*” | P001 |
|  |  |  | “*an important thing is* … *designing the time-based trajectory of use, what is the duration of the intervention? How many conversations do I expect people to have? How long will the conversations last? How frequently will people be interacting with* [the CA]? *both, ideally minimum and maximum.*” | P003 |
|  |  |  | “*what are the sensitive words we can use here to engage. Because in conversational agents, we need to be very concrete, very short dialogues but with a lot* [of information].” | P012 |
|  |  | Evidence-based content | “…*how to say things in a way that a seasoned clinician would do it. So that's like the best practices, not only is the content best practice but the way of saying it is the best practice.*” | P009 |
|  |  | Emergency and error management | “*having maybe a list of … scenarios, maybe where you might need to escalate the situation … different situations that might occur and how you would deal with that.*” | P007 |
|  |  |  | “*if the patient* … *develops new symptoms of some kind, if* [the patient] *think*[s] *the agent is close to a person, and a person would recognize that* [the patient] ha[s] *new symptoms but the agent doesn't, that's … a problem. So, it's managing errors; errors are mistakes,* [and] *also managing adverse events, or side effects or just things that happen to people that the agent may or may not, usually would not recognize unless there's intentional design to do that.*” | P009 |
|  |  | Personalization | “*personalization is so critical because that's what makes it a conversational agent ... the dialogue has to be tailored specifically for the person. And what I think makes conversational agents, conversational agents, is that is that they are able to create sentences in real time that are tailored, so it's not a videotape.*” | P009 |
|  |  | Defining the development platform | “*development doesn't include just dialogue and content; you may have other parts to be developed potentially, depending on the choice of* [platform], *whether you will develop* [the CA] *from scratch or you will use some platforms, the Alexa skills or Juji.*” | P001 |
|  |  |  | “*setting up the development environment … which tools we would use as a team? How we would coordinate the development, actually like development chatbot, which kind of testing tools we needed, where we would upload the code*.” | P004 |
|  | Evaluation and Implementation | Evaluation is iterative | “… *everyone simultaneously develop and test all the time … and then have like one large testing period.*” | P004 |
|  |  |  | “*part of the development process should be iterative changes in the system as feedback has happened*” | P009 |
|  |  | Usability | “*Either user experience is quantified into a usability framework, or usability is one dimension of user experience. They're not separate.*” | P003 |
|  |  | User engagement | “*So, there's efficacy, put the system in front of patients, does it change their health behavior or understanding or some measurable clinical outcome? First importance. But then there's the process measures, are people happy with it, satisfied with it? Are they using it, (how) is their engagement? Efficiency* [how fast the intervention is delivered] *is typically not a concern except as it impacts the other measures.*” | P003 |
|  |  | Technical evaluation | “*are there any additional things that we have to assess from a technical perspective before we run pilot studies and randomized controlled trials? Because these are very expensive when we enroll patients*” | P002 |
|  |  | Economic evaluation | “*that might be a broader perspective* [than] *just looking at economics. Whether it is desirable, whether it is feasible and whether* [it is] *viable. Desirability, I think, for humans, feasibility for the technology, viability for the organization.*” | P001 |
|  |  | Implementation | “*Using the results from the evaluation to inform you know the next decision to the implementation.*” | P007 |
|  |  |  | “*implementation, meaning taking an evidence-based practice and testing it in the real world outside an RCT*.” | P009 |
|  |  |  | “*Implementing a new process in a health system is really hard to do because it's a complex environment, and you have to involve clinicians or at least hospital staff, both in the development and the implementation.*” | P009 |
|  | Cross-cutting themes | User-centered design | “*user-centered design is a process, but the privacy and security is more like a design consideration*” | P001 |
|  |  |  | “*it's just a matter of reformulating, not call it user-centered design… it's maybe that's just user involvement, and it might be the same level as data privacy and security*” | P002 |
|  |  |  | “*it's time consuming because you probably need to conduct a study, a qualitative study, but I think it's worth it, of course. I see the difficulty that different people* [may] *provide different information, especially in terms of conversational flow.*” | P006 |
|  |  |  | “*It's still important to find out what they want, but sometimes it's better to do this in an experimental setting rather than just asking people*” | P008 |
|  |  | Data privacy and security | “*Where will the conversation*[al] *agent store user data? … is this an online system that is requiring Internet access, or is it offline? … Encryption of data*” | P002 |
|  |  |  | “*in design, you're thinking about requirements, development, you're implementing them, and then in evaluation, you're not only concerned about data in the system, but your outcomes measured data and how you're handling it for analysis… So, that seems like a reasonable cross cutting issue.*” | P003 |
|  |  |  | *“if* [an] *app is going to be very extensive, uses a lot of data, and* [privacy is] *an important part of it,* [the researcher] *should explain about the data. And if* [it is] *a more surface app … for example, a passive exercise collecting app, then maybe it's not so necessary.”* | P008 |
|  |  | Ethics | “*ethics, I think, missing in this conceptual framework, those kind of overarching dimensions, and then you have a more processed view.*” | P001 |
|  |  |  | “*I don't see ethics mentioned anywhere here, and ethics really should be central … there's user-centered design, data privacy and security, which does relate to ethics some bit, but maybe another third category that could be ethics so, it would be ubiquitous that way.*” | P005 |
|  |  | Long-term sustainability | “*where* [the CA] *fits in the bigger health system.*”  “*it probably would be better if* [the CA was] *designed thinking about, once it was not a research project, where it fits, how are people* [going to] *know about it? Who's going to encourage people to use it?*”  “*Yes, sustainability*” | P010 |
